# Through Ancient Rings Thread Programming Strings

**DOI:** 10.1016/j.str.2009.10.006

**Published:** 2009-11-11

**Authors:** Martyn F. Symmons, Ben F. Luisi

**Affiliations:** 1 Department of Biochemistry, Tennis Court Road, Cambridge, CB2 1GA, UK; 2 Department of Pathology, Cambridge, CB2 1QP, UK

## Abstract

A new crystal structure of assembled subunits from the eukaryotic exosome complex gives insight into the interactions underpinning its various functions (Bonneau et al., 2009). Here, we focus on what the emerging structures tell us about the regulation of the exosome interactions with, and actions on, RNA.

## Main Text

If a parallel may be drawn between the processes of life and of computation so that DNA is considered the imperfectly replicated template of inherited programs, then what do we make of RNA? Beyond its roles in translation as short-lived intermediary and component of the protein-synthesis machinery, RNA moonlights as a key regulator of gene expression. In its noncoding capacity, RNA boosts astronomically the information content of the DNA-encoded program. With so much RNA about that can impact the computational program, it is imperative that all RNAs—coding as well as noncoding—are maintained at appropriate levels, that the information they confer lasts only as long as needed and no longer, that their quality is controlled, and that they are efficiently scavenged for recycling at the end of their useful lifetimes.

Perhaps unsurprisingly, several biological machines have evolved to meet the exacting demands for RNA turnover, surveillance, and precise maturation of structured precursor RNAs (Houseley and Tollervey, 2009). One such machine that plays multi-faceted roles in RNA catabolism is the exosome. Found in archaebacteria and both the nucleus and the cytoplasm of all known eukaryotes, the exosome is a multi-enzyme assembly that can interact functionally with partner assemblies to participate in diverse processes, such as the degradation of defective RNA or the rescue of stalled ribosomes. Accumulating structural data have provided insights into exosome function and evolution. In a key advance reported in *Cell*, Bonneau et al. (2009) now show how the exosome subunits bring a range of binding and catalytic domains to bear on substrate RNA.

**Figure 1:**
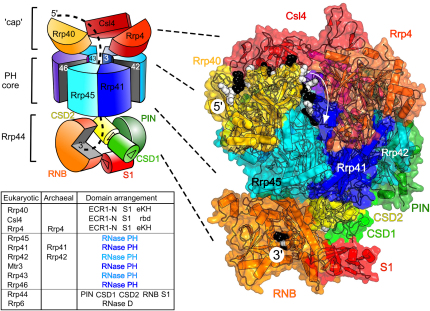
The Arrangement of Rrp44 Domains under the Human Exosome Core, with a Hypothetical RNA Substrate A cartoon schematic of the full human exosome is shown (left) with RNase PH core of six subunits (shown as a trimer of dimers; each pair in light blue and dark blue) around the central channel. Above this is the “cap” of RNA binding subunits (orange and reds). Finally, below is the Rrp44 subunit but here with the domains indicated and colored separately (dark green through orange to red, with arrows on bases of separate domains pointing to RNA-interacting faces). A structural model of this arrangement with the same viewpoint and coloring scheme is shown in greater detail as a drawn schematic under a transparent surface (right). This is the human exosome (2NN6.pdb; Liu et al., 2006) positioned with the Rrp45:Rrp41 heterodimer over a model of the domains of complete Rrp44 now revealed by the structure of Bonneau et al. (2009). This view uses their previous structure of the RNase II domain (RNB; Lorentzen et al., 2008) with its associated RNA binding domains (CSD1, CSD2, and an S1 domain, all OB folds; http://scop.mrc-lmb.cam.ac.uk/scop) and a substrate with its 3′ end bound at the RNB exonuclease site. The structural PIN homolog (1YE5.pdb) used to complete this will now be superceded by that of the complete Rrp44 and the atomic details of its interactions (Bonneau et al., 2009). The domains (from N to C terminus) in the subunits of the eukaryotic and the much simpler archaeal exosome are listed in the table alongside the structure. ECR1-N designates the β stranded N-terminal domain of the exosome RNA binding subunits (http://scop.mrc-lmb.cam.ac.uk/scop); S1 is an OB fold, eKH is the eukaryotic type of KH RNA binding domain, and rbd is a fold, perhaps vestigial, similar to rubredoxin (http://scop.mrc-lmb.cam.ac.uk/scop). The RNase PH domain is conserved (Symmons et al., 2002) but is now known to not necessarily be catalytically active. PIN (dark green) is an endonuclease domain distantly related to RNase H; each CSD (yellow-green and yellow) is an OB fold similar to S1 and small cold-shock domains; RNB (orange) is an RNase II-like exonuclease and RNase D is also a conserved hydrolytic RNase. The structural homology of the RNase PH trimer of antiparallel dimers to the bacterial homolog PNPase allows the speculative modeling of an entrapped route for longer RNAs from the defined OB fold binding sites through the central channel (Symmons et al., 2002). This extends the Lorentzen et al. (2008) structure of the Rrp44 exonuclease complex to produce a model (shown as alternating black and white nucleotides in the model on the right and dotted lines in the schematic model on the left) of a 30-mer of single-stranded RNA following a defined route through the OB domain sites of Rrp40 and Crl4 and through the channel to the Rrp44 RNB domain active site. This 30-mer therefore stands as a measuring-stick for the RNase protection assays of Bonneau et al. (2009).

The core of the exosome is a ring formed from six nearly identical subunits that are homologs of the eubacterial ribonuclease RNase PH (Symmons et al., 2002). RNase PH shares with these subunits tertiary structural elements that are rarely found in other proteins, indicating that they must have originated by divergence from a common ancestor. The hexameric ring of RNase PH-like subunits forms a scaffold for recruitment of other exosome components. Clustered on one side of the ring are S1 and KH domains, which are also ancient RNA-binding structural units. Three of these domains associate with the RNase PH-like core to form a 9-subunit core (Figure 1). Remarkably, the subunit organization of the 9-subunit core is very similar to the organization of subdomains seen in the homotrimeric bacterial enzyme polynucleotide phosphorylase (PNPase), in which the corresponding subunits are fused into one continuous polypeptide (Symmons et al., 2002). The architecture of the hexameric ring supports a central channel that is sufficiently wide for single-stranded RNA, and a similar channel is observed in the RNase PH hexamer and the pseudohexameric eubacterial PNPase. Structural studies of archaeal exosomes and eubacterial PNPase suggest that the RNA might thread through the channel, with the 3′ end of the RNA entering catalytic pockets off this channel where it is cleaved by a phosphorolytic mechanism; i.e., using inorganic phosphate as the nucleophile rather than water (Lorentzen et al., 2007; Navarro et al., 2008; Nurmohamed et al., 2009). It came as a great surprise that the RNase PH-like subunits of the eukaryotic exosome have entirely lost this catalytic capacity (Dziembowski et al., 2007; Liu et al., 2006). Instead, the ribonuclease activities of the eukaryotic exosome are provided by subunits that associate with the periphery of the 9-subunit exosome core (on the opposite surface from where the S1 and KH domains of cap subunits are bound [Schaeffer et al., 2009]; see Figure 1). These ribonuclease subunits use a hydrolytic mechanism rather than a phosphorolytic mechanism. Although the core of the eukaryotic exosome is catalytically inert, it's clearly still important, as deletion of any of the corresponding genes is lethal. What might be that essential function?

Bonneau et al. (2009) now reveals, in atomic detail, how, despite lacking enzymatic activity, the eukaryotic exosome core is likely to be used for substrate channeling. RNA channeled from the S1/KH face of the core through the central channel, as a single strand, would exit on the opposite face to meet the peripheral ribonuclease subunits, as previously suggested from careful EM reconstructions (Wang et al., 2007). The nuclease subunits bound to this face of the exosome cleave the RNA using combined processive and distributive mechanisms. In the same report, Bonneau et al. presents the structure of a portion of the RNase PH-like core engaged to the peripheral ribonuclease, Rrp44. This structure is also the first tangible proof that the human Rrp44 does indeed associate with the core exosome, as was previously demonstrated for the yeast complex (Schneider et al., 2008; Schaeffer et al., 2009). This hydrolytic ribonuclease carries a distributive endonuclease sub-domain (the “PIN” domain) and a processive exonuclease domain, and the report provides the first view of the entire Rrp44, including the key elements of this PIN domain. It was recently shown that recruitment of Rrp44 to the exosome modulates its activity (Schneider et al., 2008), and this is accounted for through conformational adjustments that accompany the subunit-subunit interaction. The role of conserved cysteine motifs in the very N-terminal regions of the PIN domain (Schneider et al., 2008) remains unknown.

One puzzling finding is that the PIN endonuclease subdomain of Rrp44 is exposed to the solvent on the exosome surface, while the exonuclease domain is orientated to intercept the end of any RNA threaded through the central channel of the core. Bonneau et al. (2009) also show that a threading mechanism for the eukaryotic exosome is supported by the effect of mutations of conserved positively charged residues at both the end and the central region of the channel. Using a modeled substrate as a measuring-stick (30 black and white nucleotides; Figure 1) shows how this threading through the exosome would give protection to the length of RNA observed in the degradation experiments. The spatial colocalization of the endo- and exonuclease activities may permit alternate routes to the active sites and cooperation between the two catalytic centers (Wang et al., 2007; Schaeffer et al., 2009). There is precedent for analogous organization and cooperation of endo- and exoribonucleases in the bacterial RNA degradosome (Carpousis, 2007). Such colocalization could efficiently decrease the lifetime of possibly deleterious fragments generated by degradation. It could also orchestrate their activities within Rrp44 or in conjunction with other components (such as the Rrp6 component, presumably bound nearby). Drawing the analogy further, work from the van Hoof group (Schaeffer et al., 2009) indicates that the PIN endonuclease is stimulated by the 5′ phosphate end of the RNA, which also occurs for RNase E, the endonuclease of the bacterial degradosome. The activation of PIN by the 5′ end of the RNA may serve a function of concentrating nuclease attack on substrates that are already cut and destined for destruction, a role that has been suggested for a similar effect in the prokaryotic RNase E (Carpousis, 2007).

The 3′ exonuclease domain of Rrp44 is presumed to engulf the end of the substrate in the exosome (only the extreme 3′ nucleotide is visible in Figure 1), as it does in the structure of the enzyme substrate complex (Lorentzen et al., 2008). A cycle of hydrolysis can favor the binding of the new 3′ end into the active site. As hydrolysis is effectively irreversible, the cumulative effect is to ratchet the substrate along, so that the liberation of each nucleoside monophosphate pulls the substrate along the channel for the next round of cleavage. The combination of the cap subunit domains and a channel that can only accommodate a single strand could produce the unwinding of structured RNA substrates. A similar energy-driven ratchet mechanism has been proposed to account for the processive degradative activity of the eubacterial homolog of Rrp44, the hydrolytic RNase R (Vincent and Deutscher, 2009).

Data from nuclease protection experiments show that the exosome core by itself is not efficient at protecting a length of RNA until the full length of the Rrp44 is associated. There is likely to be an allosteric effect of the protein-protein interaction on one surface of the core with the cap subunits that gate the entry of the 3′ end. The domains of the cap subunits include motifs that bind RNA with defined directionality, and Rrp40 is particularly implicated in this by mutations studied by Bonneau et al. (2009). The details of the threading mechanism remain a fascinating mystery.

Tails of poly(A) are added in the nucleus to target RNA for decay. It seems that the tail might aid channeling of RNA substrates into the nuclear exosome, and therefore the length of the tail is likely a key signature to mark RNA for destruction. The nuclease protection experiments suggest that the exosome can protect a length of 30 nucleotides in a single-stranded RNA. Degradation cannot be initiated near a stable stem structure, and polymerases can add a single-stranded tail that may act as a “landing pad” for the exosome. In yeast, a major cofactor for the nuclear exosome is the TRAMP polyadenylation complex that is involved in nuclear surveillance of RNAs and RNA-protein complexes (Houseley and Tollervey, 2009). Its components include a poly(A) polymerase, an RNA-binding protein, and an RNA helicase. It is conceivable that the helicase of the TRAMP complex might serve as a translocase to prime the directional threading of the single strand into the exosome.

The question naturally arises why organisms with a nucleus have retained exosomes that look like bacterial PNPase, but have entirely lost the corresponding phosphorolytic activity? We suggest that this could have arisen to prevent the exosome from randomly adding tails to RNA. Polynucleotide phosphorylase and the archaeal exosome can work backward to add tails under conditions when phosphate is low but nucleoside diphosphates are abundant (Slomovic et al., 2008). In its behavior toward poly-adenylated RNA, the nucleus is an honorary bacterium—polyA tails lead to instability there, whereas in the cytoplasm they confer stability to transcripts and structured RNA. The abundance of nucleoside diphosphates in the nucleus would favor the backward reaction. The compartmentalization of the synthesis may have gone hand-in-hand with the loss of phosphorolytic activity of the exosome. Once evolution had set up the subunit contacts to direct the RNA down the hexamer channel, it was perhaps easier at this point to push on through to a separate hydrolytic RNase than to rewind.

The ring architecture of RNase PH, PNPase, and the exosome is truly ancient, and its conservation is a reflection of its important biological roles and just how deeply embedded it has become in the regulatory repertoire in all domains of life (Symmons et al., 2002). The embellishment and tinkering of the architecture for regulation and interaction with partners are evolutionary adaptations to match the needs for expanded genomic complexity. From the perspective of life-as-computation, RNA degradation by exosomes and other machines goes hand-in-hand with the emergent complexity associated with the regulation offered by RNA.
